# Decoding Endoplasmic Reticulum Stress on Chondrocyte Driving Osteoarthritis Development through Integrating Single-Cell and Transcriptomic Profiling

**DOI:** 10.7150/ijms.119573

**Published:** 2025-08-22

**Authors:** Zhao Zhang, Debin Cheng, Jingyi Dang, Xiaohe Wang, Hongbin Fan, Dong Liu

**Affiliations:** Department of Orthopaedics, Xijing Hospital, The Fourth Military Medical University, Xi'an, China, 710032.

**Keywords:** Endoplasmic reticulum stress, Osteoarthritis, scRNA analysis

## Abstract

**Background:** Endoplasmic reticulum stress (ERS) as a potent disease regulator has been proven to be engaged in the pathogenesis and progression of numerous disorders. Osteoarthritis (OA) is a widespread degenerative disease of the joints with chondrocyte damage as the main pathologic mechanism. However, the specific role of ERS in chondrocytes during OA development remains poorly understood.

**Methods:** Integration of single-cell RNA sequencing (scRNA-seq) and bulk RNA-seq analyses to thoroughly assess the landscape of ERS in chondrocytes from OA samples. The WGCNA and unsupervised cluster analysis were integrated to identify ERS patterns. Furthermore, we screened ERS key regulators for diagnosis and prediction of OA development by three algorithms (LASSO, Random Forest, and PPI analysis). Finally, we constructed *in vitro* OA models for validating the biological roles of the identified ERS key regulators.

**Result:** scRNA-seq analysis revealed a robust association between ERS and OA progression. Unfolded protein responses, TNFA signaling via NF-κB, and apoptosis were significantly activated in the high ERS risk subpopulation. Cellular communication analysis demonstrated markedly enhanced cell-cell interactions and signaling pathways in high ERS risk subpopulations compared to low ERS risk subpopulations. Unsupervised cluster analysis identified two ERS patterns exhibiting distinct metabolic and inflammation signaling sceneries. Additionally, we identified two key ERS regulators, IGFBP3 and S100A4, and developed a novel nomogram based on these markers, which demonstrated excellent clinical predictive and guiding capabilities. Finally, we found that suppressing IGFBP3 expression *in vitro* could maintain chondrocyte metabolic homeostasis and inhibit PERK/ATF4/CHOP cascade-mediated ERS to reduce chondrocyte apoptosis.

**Conclusion:** The present study integrated scRNA-seq and bulk RNA-seq to delve into the pathogenesis of ERS driving the progression of OA and identify ERS key regulators for OA diagnosis and therapeutic intervention.

## Introduction

Osteoarthritis (OA) is the most prevalent joint disease among middle-aged and older populations as well as one of the major causes of joint pain and dysfunction 1. As a chronic progressive joint disorder, OA is characterized by cartilage degeneration, articular cartilage surface impairment, and subchondral bone remodeling 2. Despite its gradual progression and worsening symptoms over time, there are currently no effective clinical treatments to halt or slow its pathologic progression. The pathogenesis of OA is highly complex with multiple mechanisms involved, including genetic factors, mechanical stress, metabolic imbalance, and inflammatory response 3. Chondrocytes, the sole cell type in articular cartilage which play a critical role in maintaining the metabolic balance between synthesis and degradation of cartilage 4. Cartilage senescence or apoptosis arising from various causes will lead to disruption of chondrocyte homeostasis, which in turn aggravates OA 5. Consequently, there is increasing concerns to explore the function of chondrocyte homeostasis in OA development.

Endoplasmic reticulum (ER) is a cellular organelle composed of a continuous reticulum system responsible for protein and lipid biosynthesis, apoptosis, and calcium homeostasis. ER is essential for protein folding and structural maturation, maintaining the activity and function of over one-third of proteins in eukaryotic cells 6. Under physiological conditions, the synthetic and catabolic metabolisms in the ER are in a dynamic balance, which is termed ER homeostasis 7. When the organism is in environments with hypoxia, low pH, inflammatory infiltration, nutrient deficiency, or calcium-ion imbalance, ER homeostasis will be disrupted, and protein folding function will be abnormal, resulting in the rapid accumulation of misfolded or unfolded proteins in the cell, triggering endoplasmic reticulum stress (ERS) 8, 9. To counteract ERS, eukaryotic cells have come up with a series of complementary adaptive mechanisms, including the unfolded protein response (UPR) and endoplasmic reticulum-associated degradation (ERAD) pathways. These processes aim to eliminate misfolded proteins and restore ER homeostasis 10. Both of the two intracellular adaptive responses as protein quality control programs are initiated by ERS to respond to malfunctions during protein synthesis, folding and structural maturation. Typically, the UPR is sensing ERS through three transmembrane sensor proteins located on the endoplasmic reticulum membrane, including activating transcription factor 6 (ATF6), protein kinase R-like endoplasmic reticulum kinase (PERK), and inositol-requiring enzyme 1 (IRE1), to restore ER homeostasis 11. ERAD can transport unstable proteins from the ER to the cytoplasm and degrade them through the ubiquitin-proteasome system. This facilitates the reduction of ER loading and avoids the deleterious effects of accumulation of unfolded or abnormally folded proteins on the cell 12. When ERS is persistent and aggravated, this dynamically balanced regulatory network can be disrupted leading to apoptosis 9. Thus, ERS is key to the development and progression of numerous diseases 6.

Recently, many studies have demonstrated that ERS-related proteins, including CHOP, ATF6, PERK, and IRE1, are obviously increased in cartilage tissues of OA patients, with their expression levels positively correlated with the degree of cartilage degeneration 9, 13. The strong association between the OA incidence and aging further supports the involvement of ERS in the pathogenesis of OA, as ER folding mechanisms and UPR function have been shown to decline with age 6. ERS had been proven to be tightly linked to chondrocyte apoptosis in the progression of OA 14. However, the specific mechanism of ERS signaling in the development and progression of OA remain poorly understood. Single-cell RNA (scRNA) sequencing, an emerging technology that recognizes distinct cell types and markers to explore intercellular transcriptome variation and heterogeneity, has raised new insights for understanding the pathogenesis of osteoarthritis 15.

**Scheme 1:**
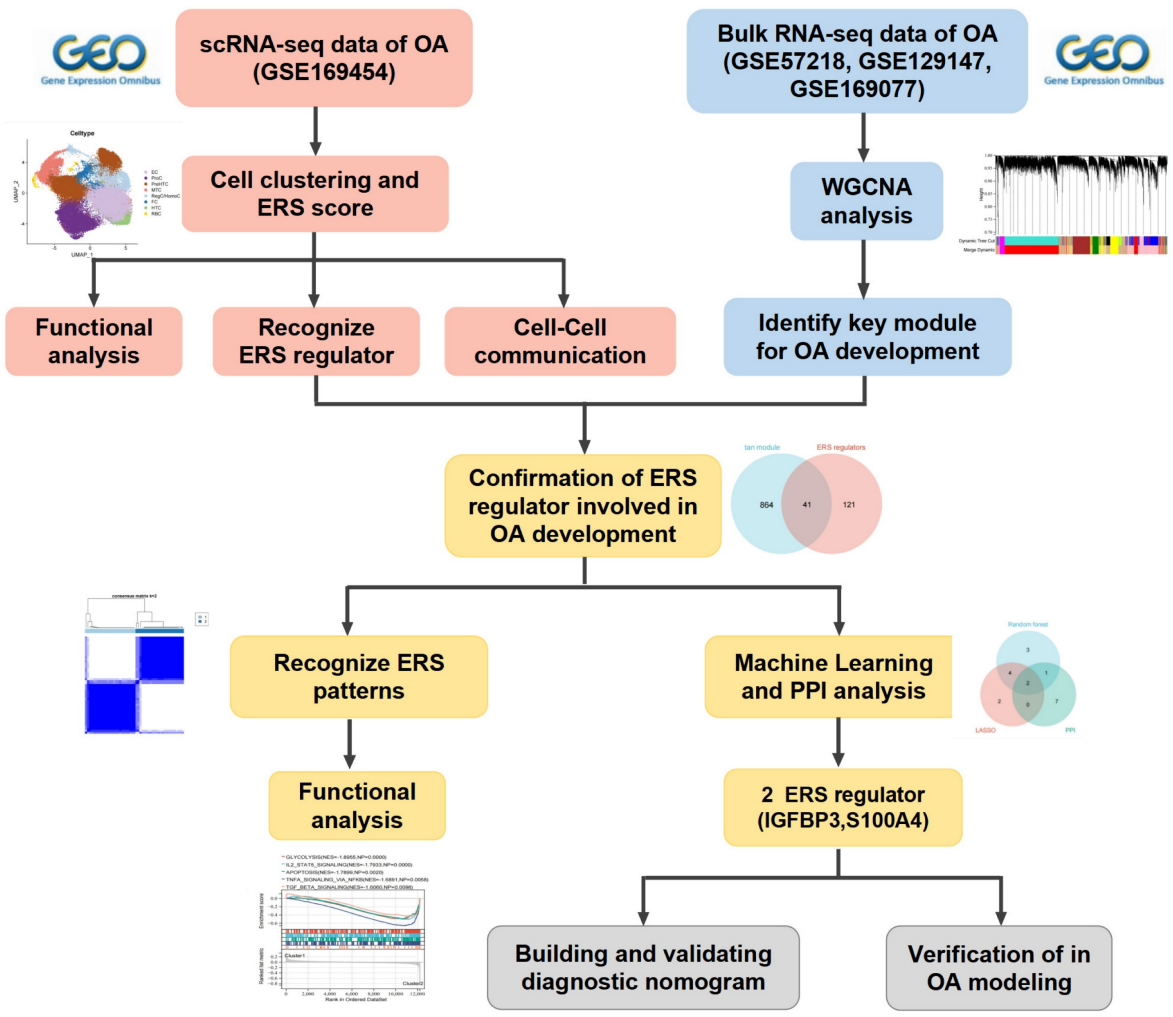
The work flow of this study.

In this study, we aimed to integrate scRNA-seq and bulk RNA-seq to explore the heterogeneity of ERS in chondrocytes and identified the ERS key regulators that are involved in the OA development (Scheme 1). Our study revealed the potential contribution of ERS in OA onset and elevated novel strategies for the diagnosis and treatment of OA.

## Methods and Materials

### Data collection and processing

To focus on the analysis of cartilage, we only selected datasets containing cartilage tissue for inclusion in this study. The scRNA-seq for OA cartilage was obtained from the GSE169454 dataset (GPL16791 Illumina HiSeq 2500) in the GEO database. The GSE169454 dataset contains four OA cartilage samples and three healthy control (HC) cartilage samples. The bulk RNA-seq for OA cartilage was obtained from the GSE57218 (GPL6947 Illumina HumanHT-12 V3.0 expression beadchip), GSE129147 (GPL15207 Affymetrix Human Gene Expression Array) and GSE169077 (GPL96 Affymetrix Human Genome U133A Array) datasets in the GEO database. GSE57218 contains 33 OA cartilage samples and 7 HC samples, GSE129147 contains 10 OA cartilage samples and 9 HC samples, and GSE169077 contains 6 OA cartilage samples and 5 HC samples.

The R package “Seurat” was utilized for the processing of scRNA-seq data. For the purpose of quality control, genes expressed in at least 5 cells were retained and cells with less than 300 or more than 7000 features were culled while retaining less than 10% of the mitochondrial reads. Subsequently, the gene expression matrix was normalized and scaled. The top 3,000 highly variable genes were identified using the FindVariableFeatures function and were used as input for principal component analysis (PCA). Shortly, the FindNeighbors and FindClusters (resolution = 0.45) functions were executed to detect cell clusters and assess robustness, and then projected through the t-SNE. The FindMarkers function was used to identify specific marker genes for each cell clusters, and heat maps were used to demonstrate the presence of significantly differentially expressed genes between cell clusters to confirm the biological validity of the clustering. Principal cell types were manually annotated based on established marker genes from the literature 16-18. R package “limma” was applied to the processing and integration of the batching effect on bulk RNA-seq. For integrative analysis of different datasets, we first extracted the gene symbols common to multiple datasets and then labeled the different datasets as different batches, again using the removeBatchEffect function to remove the batch effect. Principal component analysis was used to compare the effects before and after batch removal. Subsequently, we constructed a weighted gene co-expression network using the R package “WGCNA” to screen potential genes associated with the OA development. The most suitable soft threshold for WGCNA was determined to be 6. Subsequently, the adjacency matrix was converted to a topological overlap matrix (TOM) and the modules were defined as branches of a hierarchical clustering tree. Pearson correlation analysis was performed to identify the modules most relevant to OA occurrence.

### Evaluating ERS Scores in scRNA-seq for OA

Based on previous literature, we obtained the GO RESPONSE TO ENDOPLASMIC RETICULUM STRESS and GO REGULATION OF RESPONSE TO ENDOPLASMIC RETICULUM STRESS from Molecular Signature Database (MSigDB) v7.5 database acquired 295 ERS-related genes (Table S1) 19, 20. Based on the expression of ERS-related genes, we calculated the ERS score for each cell using R package “AUCell”. Subsequently, on the basis of the median ERS score, each cell of OA was categorized into a high ERS risk and a low ERS risk. Differentially expressed genes (DEGs) between different ERS cell subpopulations based on the “FindMarkers” function of the “Seurat” program, these genes were considered as ERS regulators of OA.

### GSEA, GSVA and cell-cell communication analysis

The KEGG and hallmark gene sets, which summarize and represent specific well-defined biological states and functions, were downloaded from the Molecular Signatures Database (http://software.broadinstitute.org/gsea/msigdb). GSEA analysis was performed based on the rank ordering of ERS regulators, with |NES|>1.5 and a P value < 0.05 considered statistically different GSVA was performed using the R package “GSVA” to characterize differences in cellular pathways. R package “cellchat” was employed to predict signaling inputs and outputs and cellular functions among different cell subpopulations.

### Recognizing ERS patterms in patients with OA

For the intersection of genes identified by scRNA-seq for ERS regulators and WGCNA for module genes related to OA occurrence, we performed unsupervised cluster analysis using the R package “ConsensusClusterPlus”. Consensus Matrix, Cumulative Distribution Function (CDF), and Tracking Map were used to determine the optimal number of clusters.

### Recognition of ERS key regulators

To identify key ERS regulators involved in OA, we integrated 2 machine learning algorithms (LASSO and Random Forest) with PPI analysis 21, 22. The LASSO algorithm reduced the number of dimensions by using the R package “glmnet” and the minimum lambda value was used as the threshold value. The Random Forest algorithm uses the R package “random forest” to filter the candidate genes with a relative importance score > 1.0 as the threshold. A PPI visualization network was constructed based on cytoscape, and hub genes were identified using CytoHubba's degree algorithm. To enhance the robustness of the results, the intersection of the three algorithms was identified as ERS key regulators in OA.

### Diagnostic performance evaluation and predictive nomogram

The R package “pROC” was utilized to evaluate the diagnostic performance of ERS key regulators. Then, nomogram was constructed to predict the occurrence of OA based on the R package “rms”. Calibration curve and clinical decision curve (DCA) were utilized to evaluate the predictive performance of the constructed nomogram.

### Chondrocyte isolation and culture

Chondrocytes were obtained from the knee joints of 2-week-old male Sprague-Dawley (SD) rats at the Animal Experiment Center of the Fourth Military Medical University. All experiments were approved by the Animal Ethics Committee of the Fourth Military Medical University (IACUC-202356281). Chondrocytes were obtained through overnight digestion by type II collagenase. Chondrocytes were then expanded and cultured in DMEM with 10 % fetal bovine serum (FBS) supplemented with 1 % P/S (v/v) to P2 for subsequent experiments.

### Small interfering RNA (siRNA) transfection

According to the manufacturer's instructions (GenePharma Biotech, Shanghai), IGFBP3-targeted siRNA and negative control (NC) were mixed with Lipofectamine 3000 transfection reagent and Opti-MEM medium. The mixture was then added to chondrocyte medium for transfection (siRNA concentration of 50 nM). The medium was replaced with normal medium after 24h of co-culture. Transfection efficiency was confirmed by real-time fluorescence quantitative PCR.

### qRT-PCR

The RNA was extracted from rat chondrocytes using TRIZOL reagent and reverse transcribed to cDNA using a synthesis kit (Takara, China). qRT-PCR was performed using the BioRad CFX96 Real-Time PCR system (Bio-Rad, USA) and TB Green Premix ExTaq II (Tli RNaseH Plus). GAPDH was used as an internal reference. The primers were shown in Table S3.

### Cell viability analysis

Cell proliferation viability was determined by Cell Counting Kit-8 assay (CCK-8, Beyotime) at 1, 3 and 5 days after transfection. Briefly, 10 μl of CCK-8 solution was added to the culture medium and further incubated for 2 hours. The absorbance at 450 nm was measured by enzyme marker.

### Immunofluorescence (IF) analysis

The chondrocytes were divided into four groups: (1) control group: untreated, (2) IL-1β group: treated with 10ng/mL IL-1beta for 24 hours, (3) IL-1β+siNC group: chondrocytes transfected with si-NC were treated with 10ng/mL IL-1beta for 24 hours, (4) IL-1β+si-IGFBP3 group: chondrocytes transfected with si-IGFBP3 were treated with 10ng/mL IL-1beta for 24 hours. Chondrocytes were fixed with 4% paraformaldehyde for 30 minutes and the membrane was broken with 0.2% Triton X-100 for 15 minutes. To block nonspecific binding, cells were incubated with 5% BSA for 2 hours at room temperature. After the closure step, cells were incubated with primary antibodies against MMP13 (proteintech, 18165-1-AP, China, 1:100) and COL II (proteintech, 28459-1-AP, China, 1:100), at 4°C overnight. Afterwards, incubate with secondary antibody for 2 hours in the dark and stain the nuclei with DAPI for 5 minutes. Observation and capture of results using fluorescence microscopy.

### Western blot (WB) analysis

WB analysis was performed as previously reported 23. Total protein was extracted from chondrocytes using RIPA lysis buffer (Solarbio, China). Extracted proteins were quantified by BCA protein assay kit (Solarbio, China). Subsequently, after electrophoresis, transmembrane, and closure, it was treated with anti-PERK (Affinity,1:2000, AF5304, China), p-PERK (Affinity, 1:2000, DF7576, China), IGFBP3 (proteintech,10189-2-AP, China, 1:2000), ATF6 (proteintech, 24169-1-AP, China, 1:2000), ATF4 (proteintech, 10835-1-AP, China, 1:2000), CHOP (proteintech, 15204-1-AP, China, 1:2000), GRP78 (proteintech, 11587-1-AP, China, 1:2000), BAX (proteintech, 50599-2-AP, China, 1:2000),and cle-Caspase3 ((proteintech, 19677-1-AP, China, 1:2000) at 4 ºC overnight. The following day, the NC membrane was incubated with secondary antibody (1:2000) for 1 hour at room temperature and observed on the ECL system.

### Statistical analysis

Statistical analysis was performed using SPSS 22.0 (IBM, Chicago, USA). All quantitative variables are expressed as mean ± standard deviation (SD). Student's t-test or one-way analysis of variance (ANOVA) was used to compare differences between groups. p<0.05 indicates a statistically significant difference (*p<0.05, **p<0.01, and ***p<0.001).

## Result

### Single-cell profiling of chondrocytes from HC and OA patients

**Figure 1:**
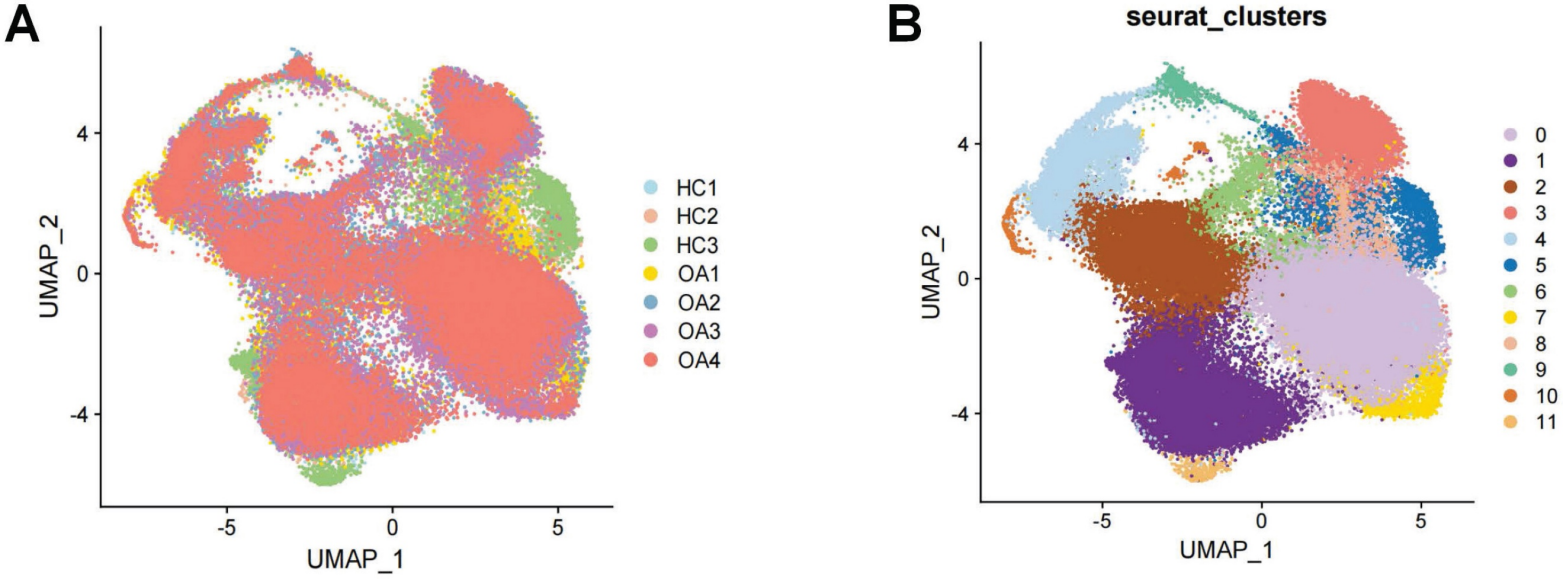
Integration of OA and HC scRNA-seq sample. (A). Cartilage samples from 3 HC and 4 OA patients; (B). T-SNE plots of 12 clusters.

**Figure 2:**
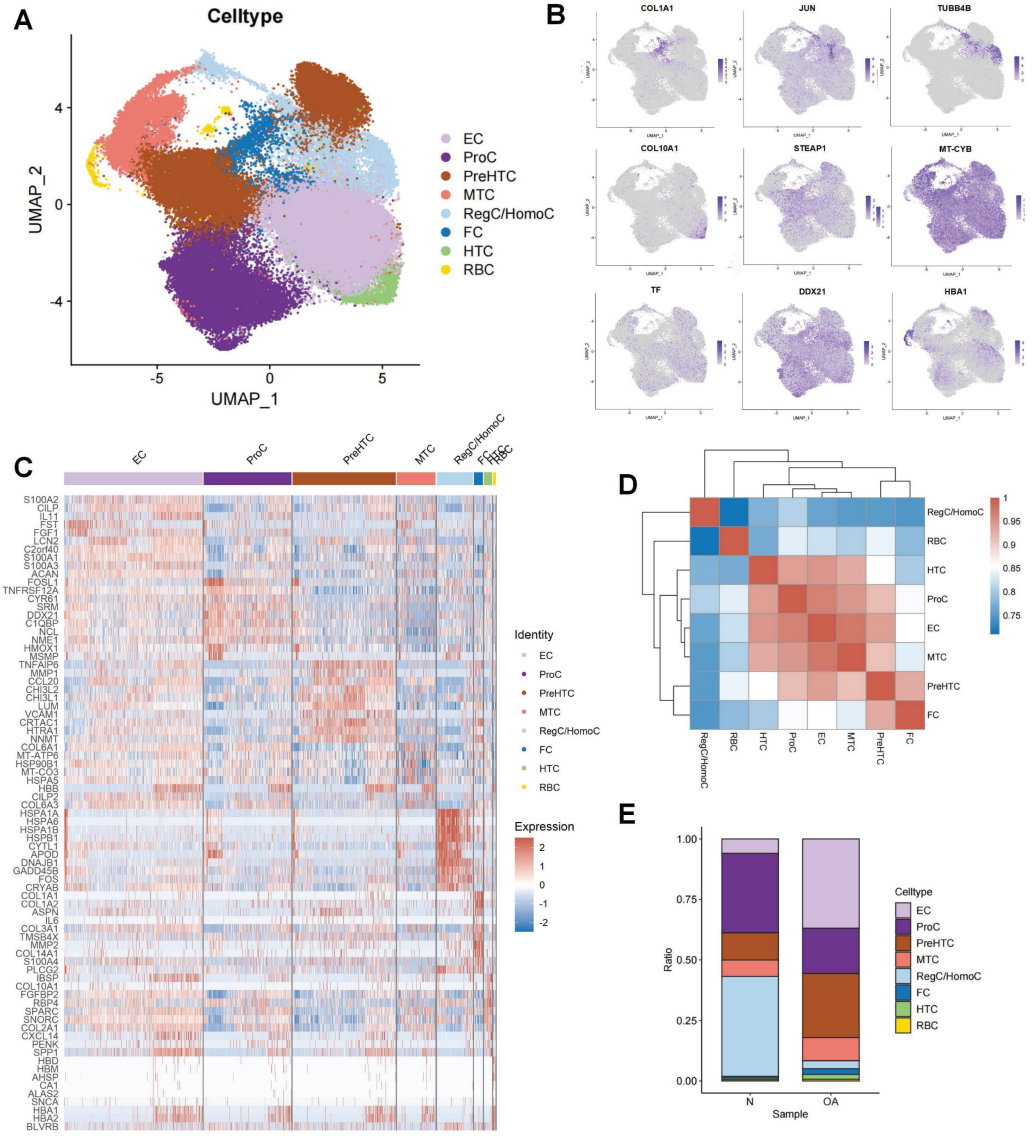
Cell subpopulation identification by scRNA-seq.(A). UMAP plot of cartilage samples; (B). Marker genes for different subpopulations with UMAP; (C). Heatmap of markers for each subpopulation; (D). Heatmap of correlations for different subpopulations; (E). Bar chart of subpopulation proportions for HC and OA samples.

Following the initial screening of the quality control program, a total of 65,362 high-quality cells were obtained from the scRNA-seq data, including 56,335 OA chondrocytes and 9,027 HC chondrocytes. Detailed results of the pre-processing of cells and features are shown in Figure S1. A total of 12 clusters were identified after log-normalization and dimensionality reduction (Figure 1A-B). Subsequently, based on previously reported cellmarkers, we identified nine cell subpopulations, including effector chondrocyte (EC), prehypertrophic chondrocyte (preHTC), fibrocartilage chondrocyte (FC), regulatory chondrocyte/homeostatic chondrocyte (RegC/HomC), proliferative chondrocytes (ProC), hypertrophic chondrocytes (HTC), mitochondrial chondrocytes (MTC), and red blood cell (RBC) (Figure 2A). The canonical gene markers, DEGs and the relation of different cell subpopulations were shown in Table S2, Figure 2B-D and S2. In HC cartilage sample, ProC and RegC/HomoC were the predominant cell subpopulations. In contrast, there was a significant decrease in the abundance of RegC/HomoC and ProC, with EC and PreHTC accounting for the major components in OA cartilage sample (Figure 2E). This indicated a dramatic change in the microenvironment of OA, EC and PreHTC were closely related to OA development.

### Single-cell profiling of ERS risk on chondrocytes in OA

**Figure 3:**
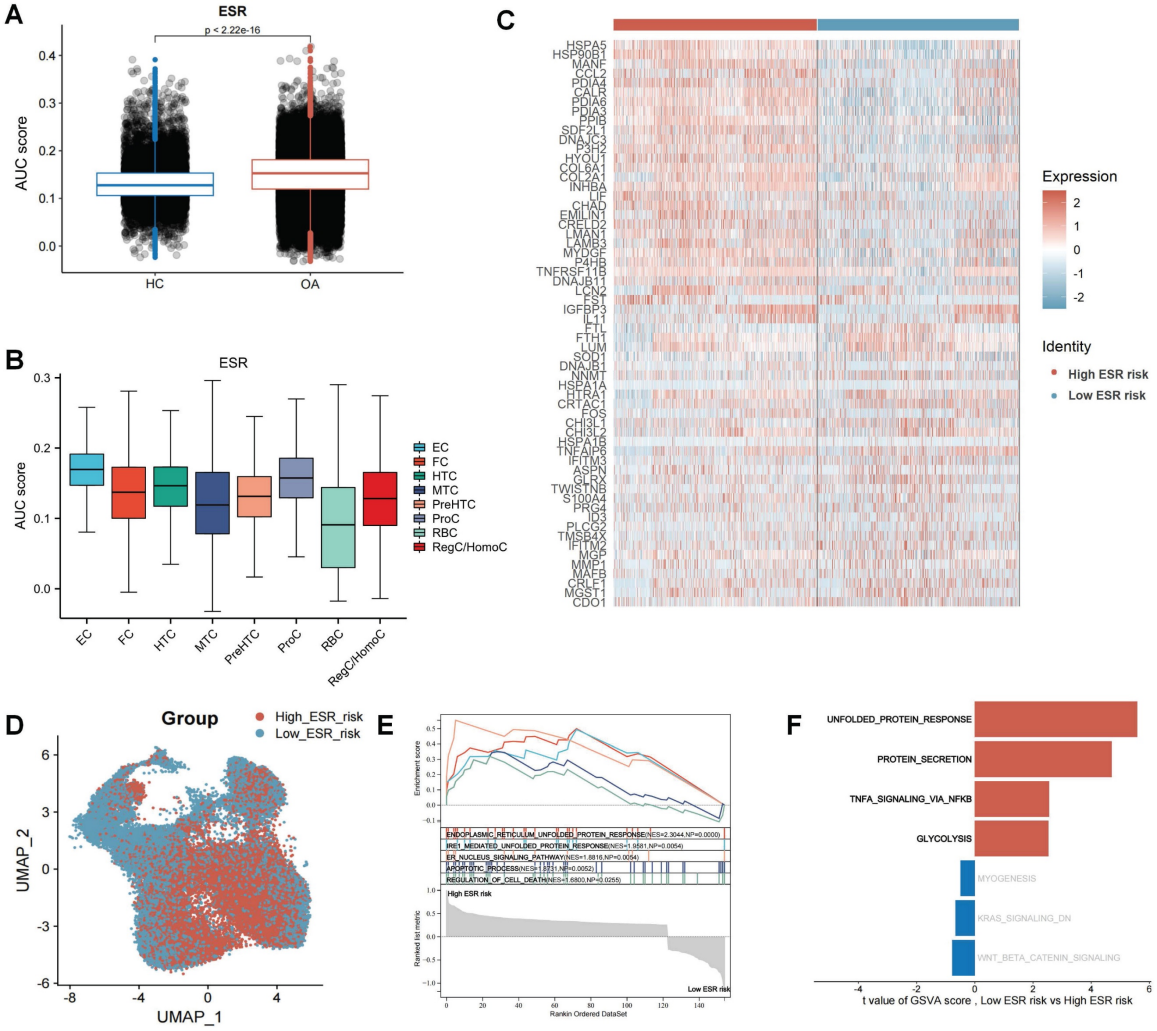
Landscape of ERS at the single-cell level. (A). Column charts for the comparison of ERS scores in HC and OA sample; (B). The level of ERS scores in different cell subpopulations in OA samples;(C). Heatmap of markers for high and low ERS risk of chondrocyte subpopulation; (D). UMAP plot of ERS risk score on chondrocyte subpopulation; (E). GSEA analysis; (F). GSVA analysis.

To reveal the effect of ERS in the development and progression of osteoarthritis, we calculated the ERS score for each cell using the AUCell. The results revealed that ERS scores were significantly higher in OA samples compared to HC samples, indicating a strong association between ERS and OA development (Figure 3A). Figure 3B illustrates the expression levels of ERS scores in different cell subpopulations in OA, with the highest scores in the EC. To reveal the heterogeneity of ERS across chondrocytes in osteoarthritis development, we categorized all chondrocytes in OA samples into high ERS risk and low ERS risk based on the median ERS score after excluding RBCs. A total of 162 differentially expressed genes were identified between the high and low ERS risk subgroups, with 121 were specifically expressed in the high ERS group and 41 in the low ERS group (Figure 3C). These genes were considered ERS regulators. UMAP plot demonstrating the expression level of ERS risk in different cells (Figure 3D). GSEA analysis revealed that endoplasmic reticulum unfolded protein response, IRE1-mediated unfolded protein response, and apoptotic process were significantly activated in the high-risk group than in low-risk group (Figure 3E). GSVA analysis showed significant activation of the unfolded protein response, protein secretion, TNFA signaling via NF-κB and glycolysis signaling pathways in high ERS risk group compared to low ERS risk group (Figure 3F).

### High ERS risk enhancing intercellular communication in OA

**Figure 4:**
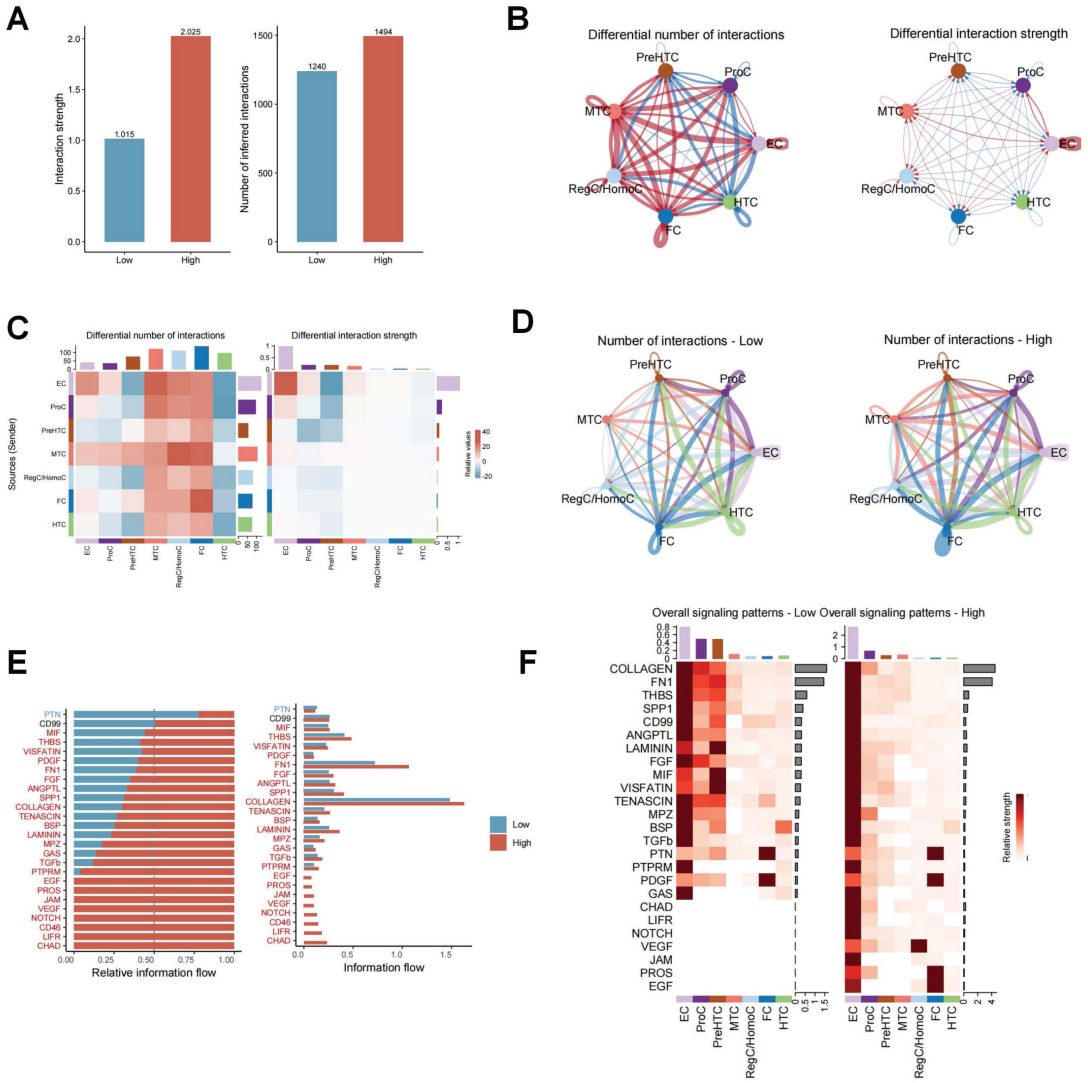
Differences in cellular communication between high/low ERS risk subgroups. (A-C). Bar plot, circle plot and heatmap demonstrating differences in number of interactions and interaction strengths in high ERS and low ERS subpopulations; (D). The number of interactions for different chondrocyte subpopulations in high ERS and low ERS subpopulations; (E-F). The variations in intercellular signaling patterns between high ERS and low ERS subpopulations.

The effect of ERS on intercellular communication was explored using the R package “CellChat”. As shown in Figure 4A, the interaction strength and number of inferred interactions were markedly higher in the high ERS risk subpopulations than in the low ERS risk subpopulations. Similar results were likewise demonstrated in different chondrocyte subpopulations (Figure 4B-D). It is indicated that ERS was involved in regulating the interactions between different chondrocytes in OA. To assess the differential expression patterns of signaling pathways in different risk subpopulations, the strength of different signaling pathways was further analyzed. The results revealed that the strength of most signaling pathways was markedly enhanced in the high ERS risk subpopulation, including CHAD, NOTCH, FN1, SPP1, VEGF, etc.

In contrast, only the PTN signaling pathway was significantly enhanced in the low ERS risk subpopulation. There was no significant difference in the activity of CD99 pathway between the two groups (Figure 4E). Figure 4F illustrated the variation in signaling pathways in different chondrocyte subpopulations. These findings indicate that high ERS risk could activate various signaling pathways to participate in the onset and progression of OA.

### Screening for ERS regulators involved in OA development

**Figure 5:**
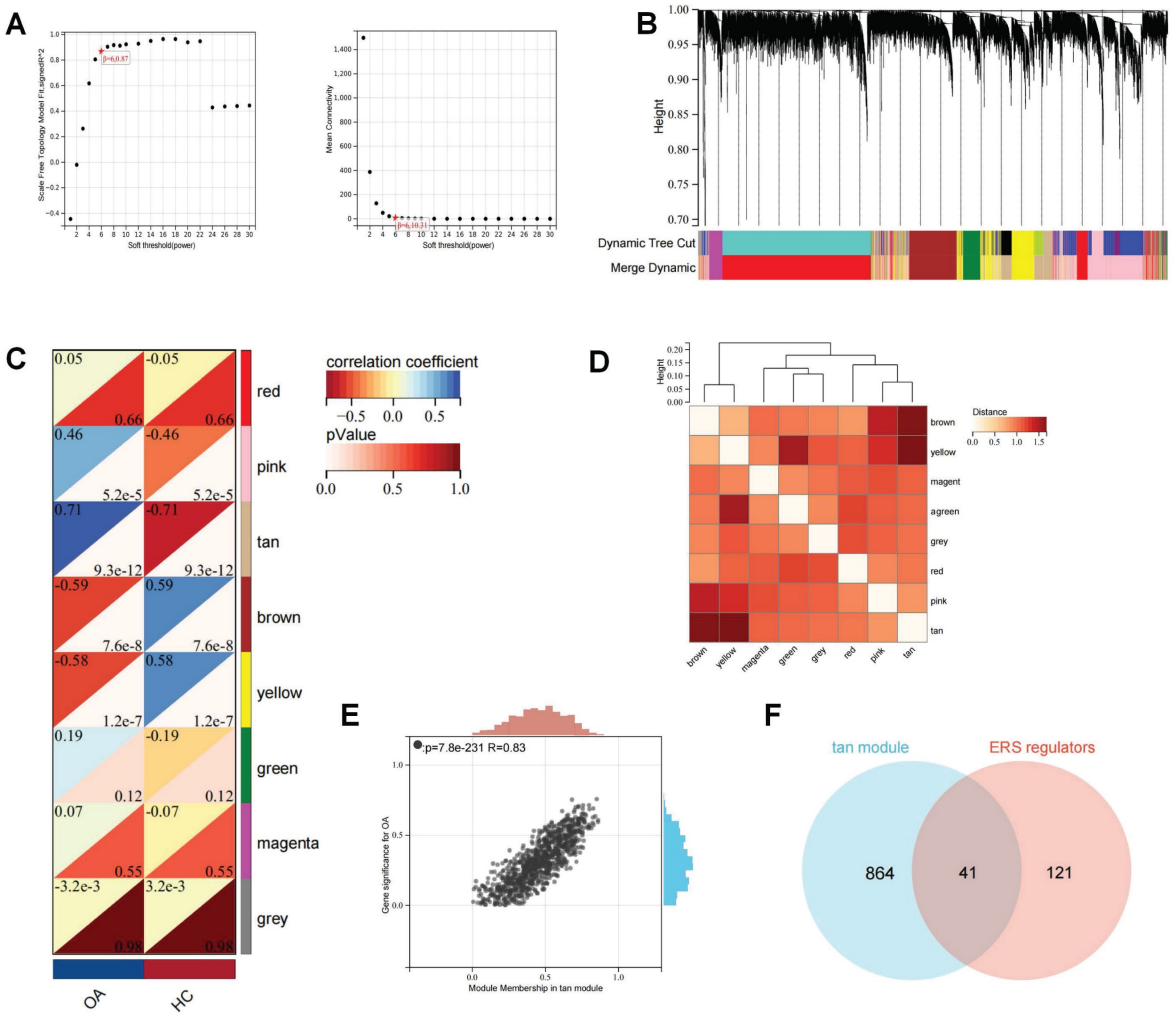
WGCNA analysis for the bulk RNA-seq of OA cartilage. (A). The selection of soft threshold of WGCNA; (B). The cluster dendrogram of co-expression genes for OA cartilage; (C). Correlation of module genes and OA clinical traits; (D). Co-relationship of different modules; (E). The significance of the tan module for OA; (F) Wayne analysis identified ERS regulators involved in OA development.

Subsequently, the batch effects of the bulk RNA-seq of the OA cartilage datasets were corrected and merged to ensure data consistency (Figure S3). WGCNA analysis was used to establish a scale-free network for OA transcriptome data. The optimal soft threshold (β= 6) was determined to achieve a scale-free topology (Figure 5A-B). Subsequently, average hierarchical clustering and dynamic tree cropping were performed to construct gene co-expression modules (Figure 5C-D). Among these modules, we found that the tan module showed the greatest positive correlation with the occurrence of OA (Figure 5E). There was a total of 905 genes in the Tan module, which were considered to be the critical genes involved in the OA development. The Wayne analysis identified 41 genes among ERS regulators and OA-critical genes that were regarded as ERS regulators involved in the development of OA.

### Characterizing the ERS pattern in OA patients

**Figure 6:**
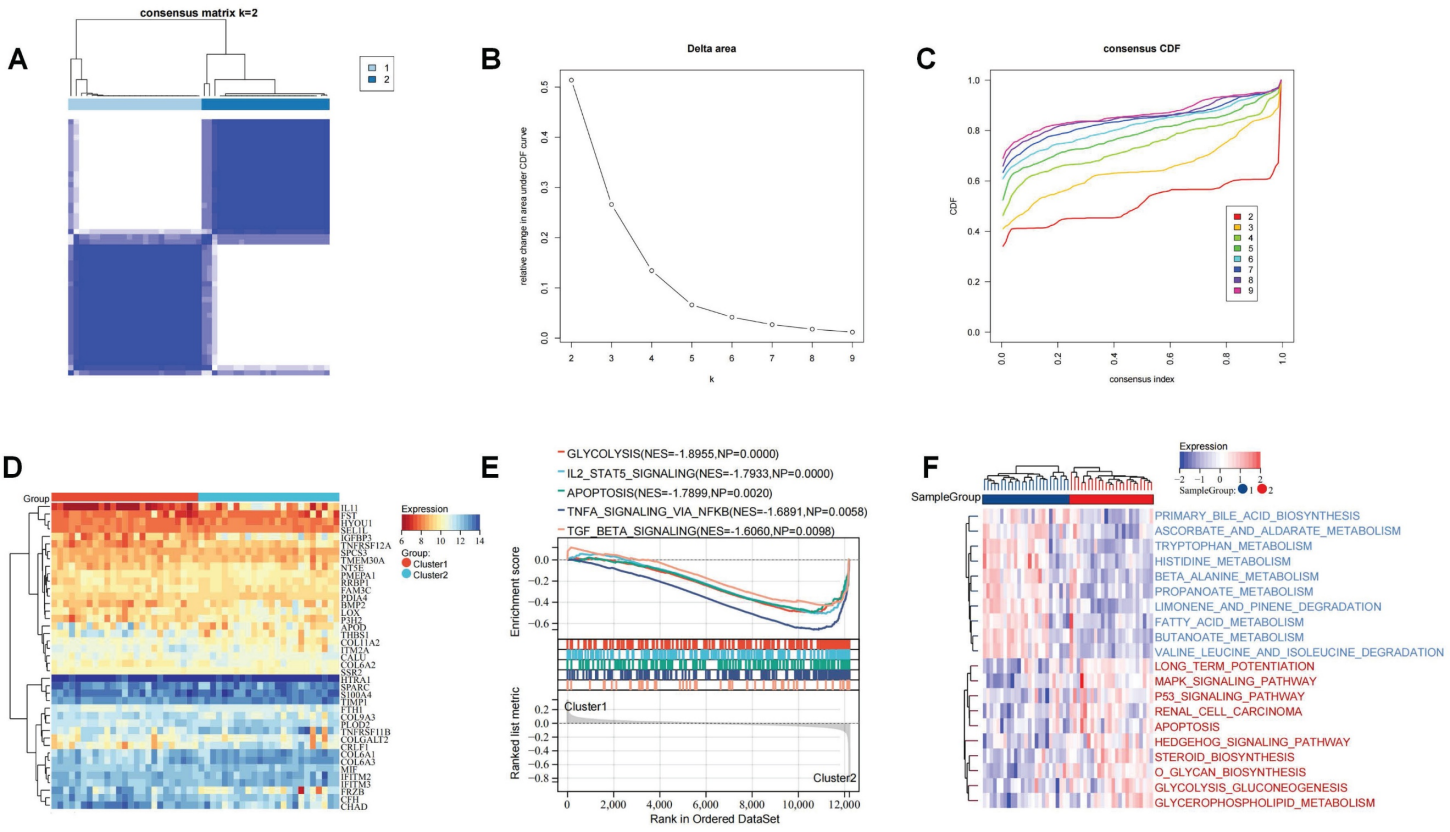
Recognition of the ERS patterns in OA. (A). Clustering matrix plot at k = 2; (B-C). CDF curve; (D). Heatmap showing the expression of 41 ERS regulators in ERS patterns; (E). GSEA analysis; (F). GSVA analysis.

To investigate the role of these ERS regulators on the development of OA, we performed unsupervised cluster analysis of the expression profiles of these 41 ERS regulators to identify ERS patterns. It was found that based on these gene expression profiles OA patients could be categorized into two distinct subgroups (Figure 6A-C). Heatmap showing the expression of ERS regulators in different patterns (Figure 6D). GSEA analysis revealed significant activation of glycolysis, IL2 STAT5 signaling, apoptosis, TNFA signaling via NF-kb, and TGF beta signaling in Cluster 2 (Figure 6E). GSVA analysis revealed that amino acid and lipid metabolism-related pathways were significantly activated in Cluster 1 patients, including primary bile acid biosynthesis, tryptophan metabolism, and fatty acid metabolism, while biochemical signaling-related pathways and glucose metabolism signaling pathways were significantly activated in Cluster 2 patients, including MAPK signaling pathway, P53 signaling pathway, O glycan biosynthesis, and glycerophospholipid metabolism. Additionally, apoptotic signaling was similarly significantly enhanced in Cluster 2 (Figure 6F). These implications mean that these ERS regulators could be used to precisely stratify OA patients via regulating the expression patterns of inflammation signaling pathways and metabolism, which raises new thoughts for personalized treatment of OA patients.

### Filtering for ERS key regulators in OA development

**Figure 7:**
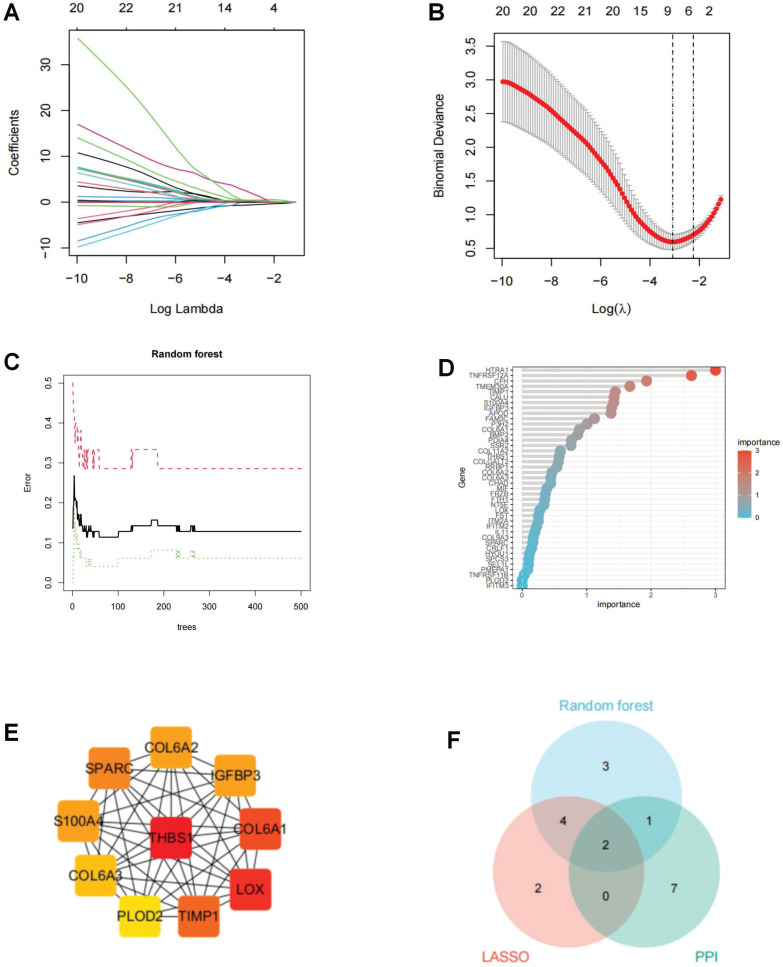
Screening of ERS key regulators in OA. (A-B). LASSO algorithm to screen ERS key regulator; (C-D). Random forest algorithm to screen ERS key regulator; (E). PPI anlysis to screen ERS key regulator in Cytohubba; (F). Wayne analysis to derive the intersection of the three algorithms.

To probe ERS key regulators involved in OA progression, we joined two machine learning algorithms and PPI analysis. The LASSO algorithm located eight ERS regulators, the RF algorithm recognized 11 ERS regulators, and the PPI analysis identified 10 ERS regulators (Figure 7A-E). To ensure the accuracy and robustness of the identified biomarkers, we performed a crosstabilization analysis of the three algorithms and identified a total of 2 ERS key regulators, IGFBP3 and S100A4 (Figure 7F).

### Diagnostic performance evaluation and prediction nomogram construction

**Figure 8:**
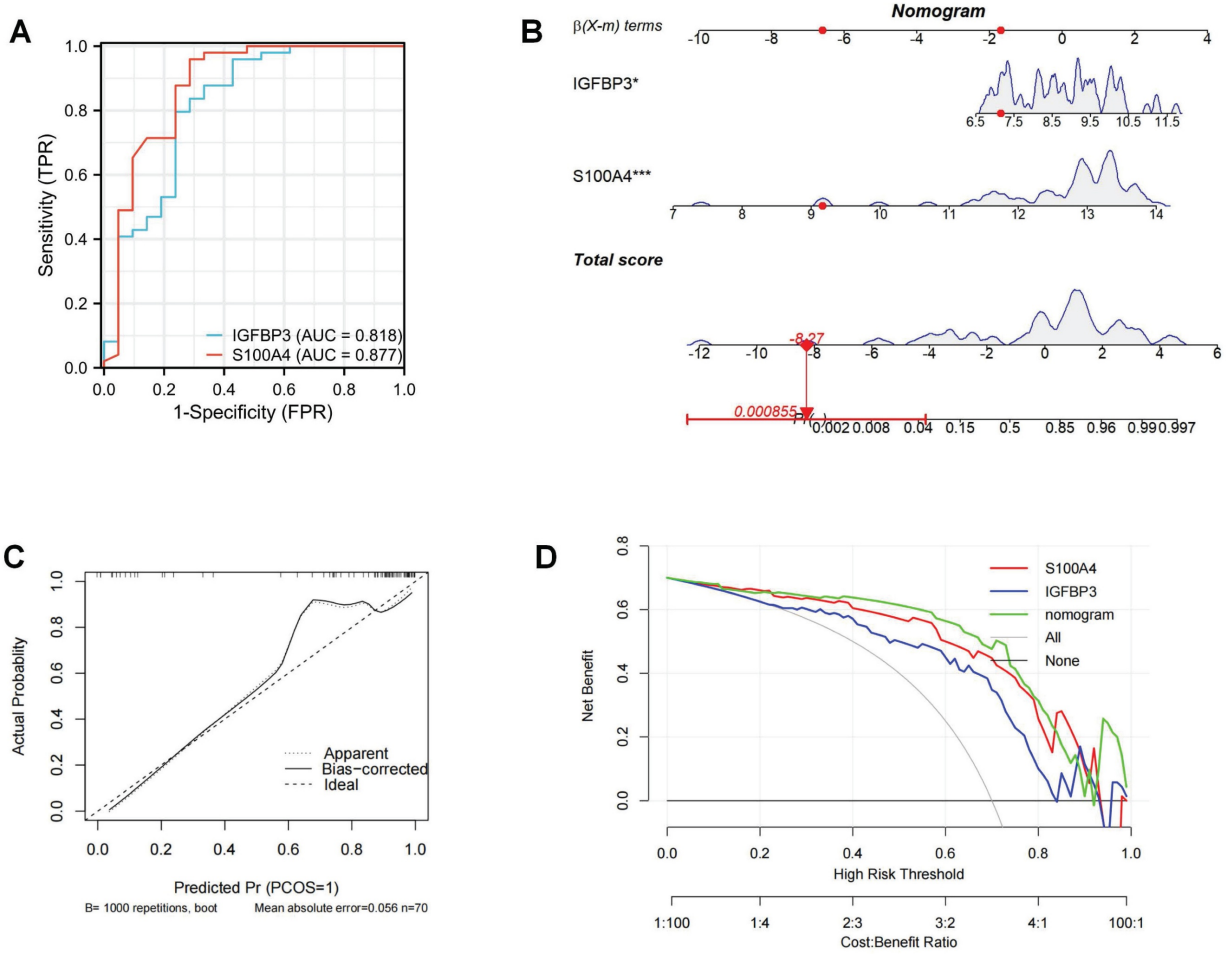
Construction of ERS risk nomogram. (A). ROC curve of ERS key regulators; (B). Nomogram to predict the development of OA; (C). Calibration curve; (D). DCA curve.

The ROC curves revealed that the AUC values of these two ERS key regulators for OA diagnosis were 0.818 and 0.877, respectively, suggesting that these two molecules exhibit a favorable OA diagnostic value (Figure 8A). To improve the prediction of the occurrence of OA, we integrated these two ERS key regulators to construct a diagnostic nomogram (Figure 8B). The calibration curve showed that the bias-corrected curve was very close to the ideal curve with a high degree of overlap. The mean absolute error (MAE) between the predicted and actual probabilities of the model is relatively small at 0.056. These indicated that the nomogram was well calibrated with good robustness and stability (Figure C). The decision curve analysis (DCA) found that the net benefit of the nomogram with a risk threshold between 0.6 and 0.8 was significantly higher than that of “None” and “All”. This suggests that the optimal clinical utility of this range is to maximize the net benefit of effectively differentiating high-risk patients and avoiding over-intervention in low-risk populations (Figure 8D).

### Single-cell profiling of ERS key regulators on chondrocytes in OA

**Figure 9:**
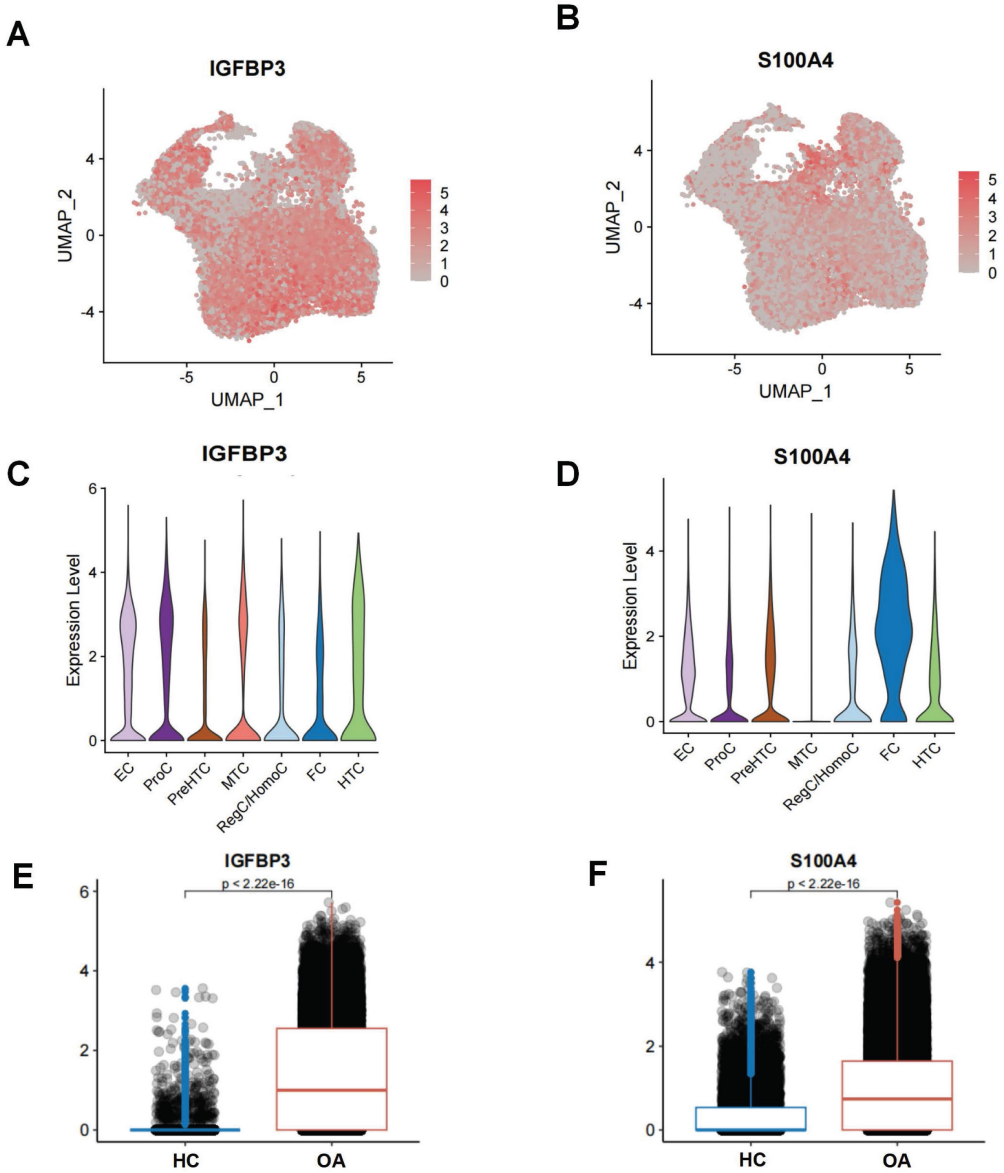
Single-cell level of ERS key regulators. (A-B). UMAP plot demonstrating the expression of ERS key regulators; (C-D). Violin plot demonstrating the expression of ERS key regulators on different chondrocyte subpopulations in OA. (E-F). Histogram showing the expression differences of ERS key regulators in HC and OA samples.

The UMAP and violin plots demonstrating the expression levels of IGFBP3 and S100A4 in different chondrocyte subpopulations in OA samples (Figure 9A-D). IGFBP3 was highly expressed in nearly all chondrocyte subpopulations, with the highest level of expression in EC, whereas S100A4 was highly expressed only in FC. Moreover, we also found that the expression of IGFBP3 and S100A4 was higher in the OA chondrocytes subpopulation than in the HC chondrocytes subpopulation (Figure 9E-F). This suggests that IGFBP3 and S100A4 might be an enhancer of ERS activation.

### IGFBP3 regulation of chondrocyte metabolic homeostasis

Above mentioned results confirm that IGFBP3 is strongly expressed in most of the chondrocytes in OA samples, which implies that IGFBP3 may not only act as an ERS driver but also contribute to the pathogenesis of OA. To further investigate the effect of IGFBP3 in the progression of OA, the cultured chondrocytes were first exposed to different concentrations of IL-1β (5,10,20 ng /mL) to mimic an *in vitro* OA model. IL-1β is a core inflammatorycytokine in the pathogenesis of OA and canreliably mimic the key pathological phenotypes of OA chondrocytes. Due to its operability, reproducibility, and stable nature, it has been widely used to simulate OA models *in vitro* 24-26.

**Figure 10:**
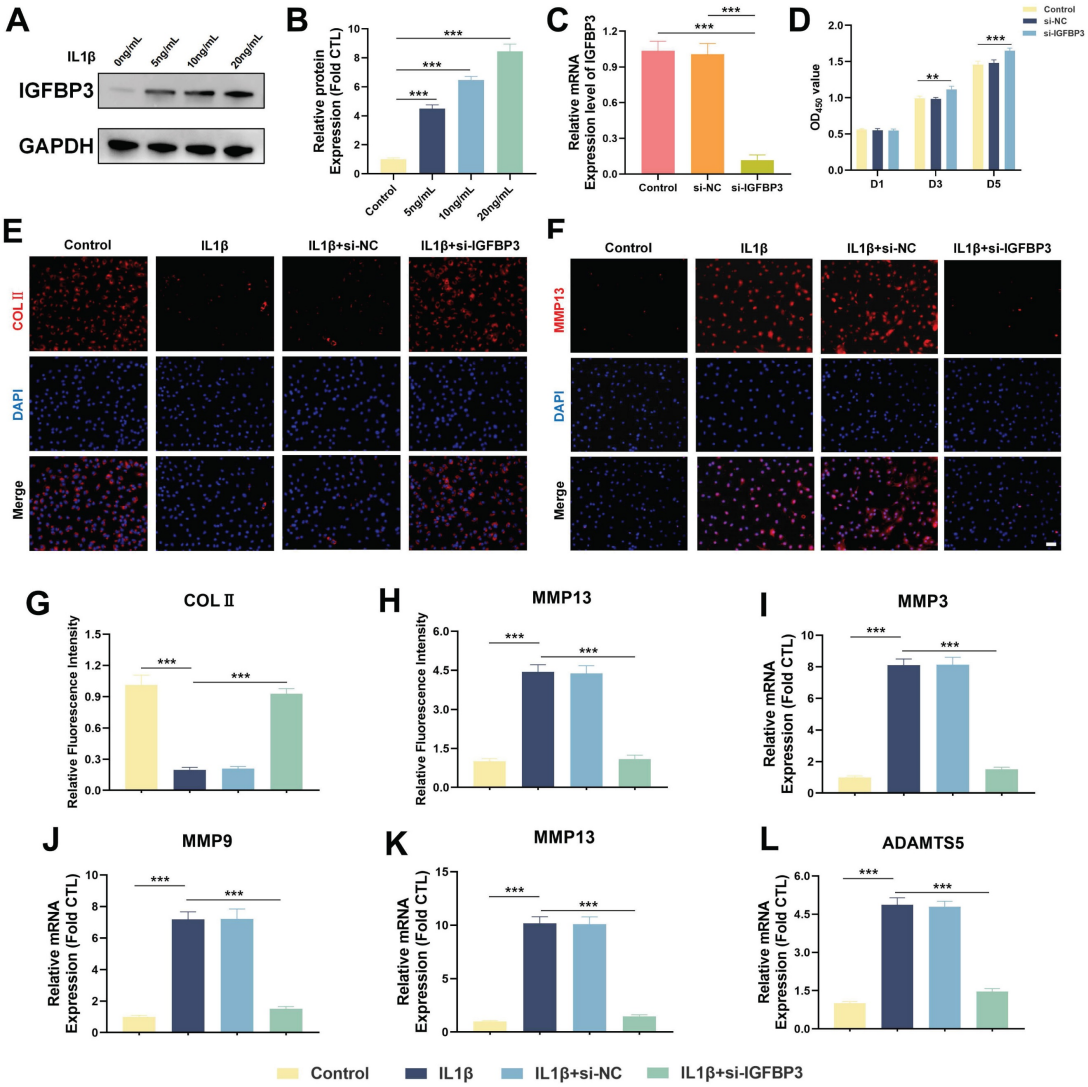
Inhibiting IGFBP3 expression can maintain chondrocyte metabolic homeostasis. (A-B). Expression of IGFBP3 induced by different concentrations of IL-1β; (C). qPCR analysis to validate the efficiency of si-IGFBP3; (D). CCK8 reagents assess the effect of si-IGFBP3 on chondrocyte proliferation; (E, G). Immunofluorescence analysis of COLⅡ expression in chondrocytes; (F, H). Immunofluorescence analysis of MMP13 expression in chondrocytes, bar=50μm; (I-L). qPCR analysis of catabolic genes (n=4, *p<0.05, **p<0.01, and ***p<0.001).

We found that the protein expression level of IGFBP3 increased in a dose-dependent manner after IL-1β treatment, suggesting that IGFBP3 is a potential pathogenic gene in OA development (Figure 10A-B). Subsequently, we used siRNA to knock down the expression of IGFBP3 in chondrocytes, and qPCR analysis demonstrated that IGFBP3 was effectively knocked down (Figure 10C). To further investigate the effect of IGFBP3 knockdown on chondrocyte viability, we used CCK-8 reagent to measure the absorbance of transfected chondrocytes at day 1, 3 and 5, respectively. Compared with untreated or si-NC chondrocytes, the proliferation activity of chondrocytes transfected with si-IGFBP3 was not significantly different at day 1, whereas the proliferative activity of chondrocytes was significantly increased at day 3 and day 5 (Figure 10D). Furthermore, immunofluorescence analysis revealed that knockdown of IGFBP3 significantly increased the expression of COL2 protein and decreased the expression of MMP13 protein in chondrocytes under IL1β induction (Figure 10E-H). qPCR analysis further assessed the expression of catabolic genes, including MMP3, MMP9, MMP13, and ADAMTS5, in chondrocytes. The results showed that the mRNA expression of MMP3, MMP9, MMP13, and ADAMTS5 in chondrocytes in the si-IGFBP3 group was significantly lower than that in the IL1β group (Figure 10I-L). These findings suggest that blocking IGFBP3 could protect the metabolic homeostasis of chondrocytes to inhibit matrix degradation in the OA microenvironment.

### IGFBP3 activates the PERK/ATF4/CHOP axis to trigger ERS to induce chondrocyte apoptosis

**Figure 11:**
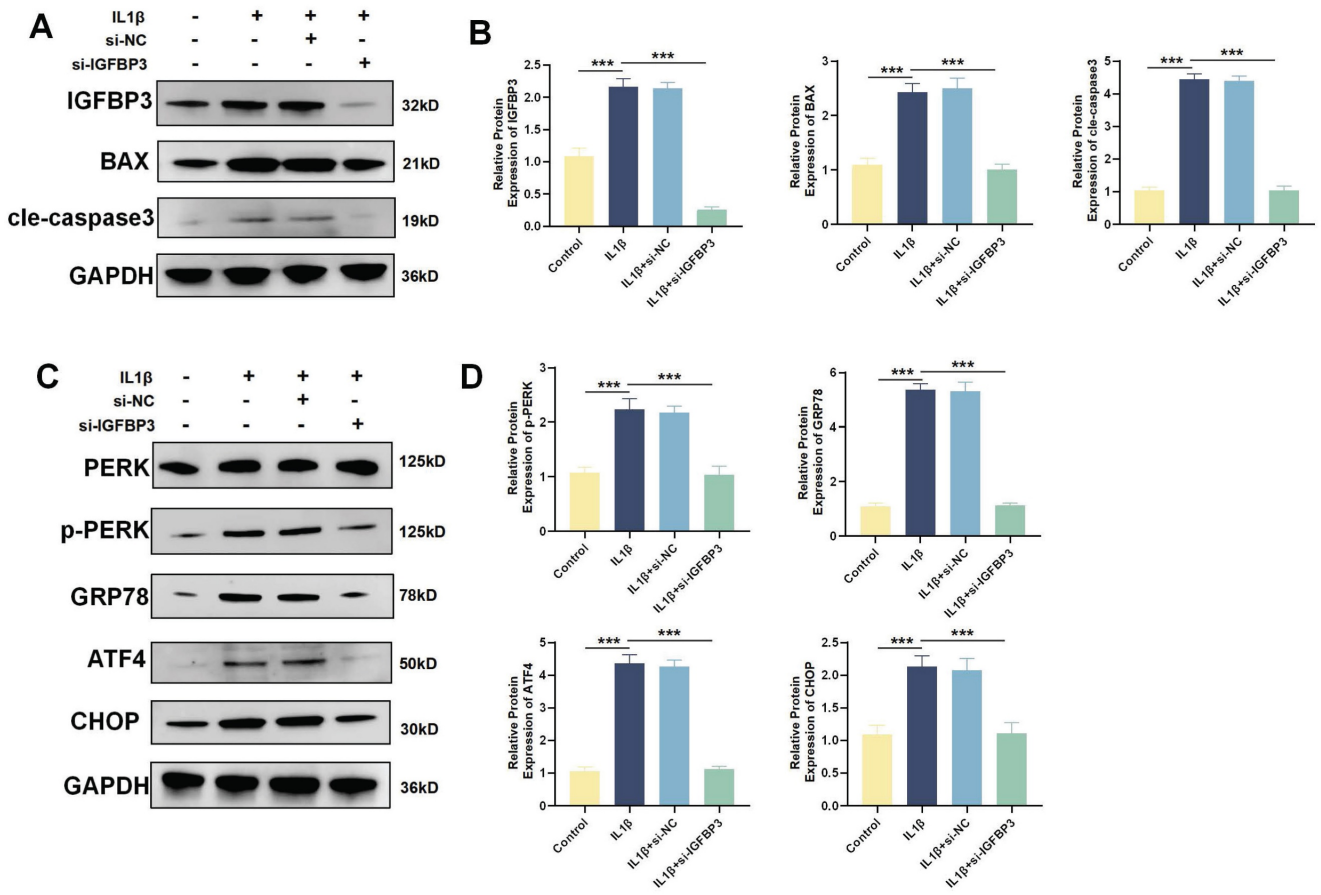
Blockade of IGFBP3 alleviated the ERS and apoptosis of chondrocytes. (A-B). WB and quantification analysis of apoptosis related protein; (C-D). WB analysis of ERS related protein (n=4, *p<0.05, **p<0.01, and ***p<0.001).

WB analysis revealed that IL1β induction increased the expression of the apoptotic proteins BAX and Cle-Caspase3. After blocking IGFBP3 expression with si-IGFBP3, apoptotic BAX and Cle-Caspase3 expression was also significantly downregulated (Figure 11A-B). When ERS is sustained, the UPR shifts from promoting cell survival to facilitating apoptosis 27. The PERK/ATF4/CHOP axis mediated ERS is intimately associated with the initiation of apoptosis 27. We found that the expression of PERK, GRP78, ATF4 and CHOP proteins was significantly increased after IL1β induction. While blocking IGFBP3 expression could significantly downregulate PERK, GRP78, ATF4 and CHOP proteins (Figure 11C-D). These suggests that targeting IGFBP3 could inhibit the PERK/ATF4/CHOP signaling cascade, thereby mitigating ERS and repressing chondrocyte apoptosis. This mechanism may help delay the onset of OA.

## Discussion

The ER is responsible for the folding of secreted proteins and the maintenance of homeostasis in the intracellular Ca2+ store 9. Protein synthesis, folding and modification in the ER are regulated precisely. These processes determine the function and survival state of the cell 7. The ERS response typically promotes cellular adaptation and survival to stress by restoring ER homeostasis, but unresolved or severe ERS can trigger cell death and mediate the onset of a variety of diseases 6. Chondrocytes constitute the sole resident cells in cartilage and are primarily in charge of regulating the synthesis-degradation balance of the extracellular matrix 6, 28. The homeostatic disruption arising from chondrocyte dysfunction or death is the key driver of cartilage degeneration and the development of OA. It has been found that ERS mainly affects chondrocytes during the progression of OA as well as being linked to cartilage degeneration. While moderate ERS can activate autophagy to protect chondrocytes from apoptosis, excessive and prolonged ERS can promote the expression of MMPs and ADAMTS5, thereby exacerbating the catabolism of extracellular matrix (ECM) and inducing chondrocyte apoptosis 11, 29. In contrast, ERS is more pronounced in synovial fibroblasts and immune cells in rheumatoid arthritis (RA) 30. Activation of the PERK/ATF6/IRE1α protein in synovial fibroblasts exacerbates the progression of RA via the promotion of inflammatory cytokine production and synovial hyperplasia 31. The ER chaperone GRP78/BiP is also present as an autoantibody in the serum or synovial fluid of patients with RA, which not only regulates the activation and function of the UPR pathway in immune cells, but also contributes to the direct production of self-antigens involved in the initiation of an autoimmune response 32. Therefore, considering the heterogeneity of ERS in arthritis, further resolving the function of ERS in OA chondrocytes is crucial for a deeper understanding of the pathogenesis of OA.

In the current study, we integrated the scRNA-seq and bulk RNA-seq to systematically investigate the landscape of ERS in OA. Based on scRNA data from OA patients, ERS was found to be significantly more activated in chondrocytes from OA patients than normal cartilage. Furthermore, ERS exhibited varying activation levels in different chondrocyte subpopulations of OA, suggesting that ERS participates in OA development and has different regulatory effects in different cell subpopulations. To delineate more definitively the complexity and pathogenic mechanisms of ERS in chondrocyte occurrence in OA, we categorized chondrocytes from OA patients into a high ERS risk subpopulation and a low ERS risk subpopulation. Functional enrichment analysis confirmed that not only UPR-related pathways were significantly activated in the high ERS risk subpopulation, but also glycolysis, TNFA signaling via NF-κB and apoptosis-related signaling pathways were significantly activated. Persistent or intense ERS can lead to extensive protein folding and synthesis, which consumes substantial amounts of energy. Glycolytic pathway activation could provide additional energy to the cell to fulfill the energy requirements of the ER in stressful states 33.

Additionally, unfolded protein-responsive transcription factors have been shown to mediate the metabolic transition from oxidative phosphorylation to glycolysis 34. NF-κB as a key regulator involved in cellular response to stimuli in chronic inflammatory diseases 35. ERS-activated UPR responses were found to promote TNF-α expression through the NF-κB pathway, thereby contributing to chondrocyte apoptosis and cartilage catabolism 36, 37. In addition, we also found significant differences in cellular communication patterns between different ERS risk subpopulations, with the signaling pathways in high ERS risk being notably more active and intense than those in low ERS risk. Collectively, these findings indicated that ERS can exacerbate the progression of OA by modulation of metabolic patterns and multiple signaling pathways, ultimately leading to chondrocyte apoptosis.

Subsequently, we further revealed the clinical guidance value of ERS expression patterns and identified key regulatory genes in OA patients using bulk RNA-seq data. Based on the results of scRNA-seq and WGCNA analysis, we identified 41 ERS regulators involved in the pathogenesis of OA. Based on the expression profiles of these genes, we categorized OA patients into two distinct ERS patterns. These ERS patterns demonstrated different functional profiles, with a large enrichment of metabolism-related pathways in Cluster 1, and aberrant activation of inflammation related pathways in addition to apoptosis signaling in Cluster 2. These further confirmed that ERS is closely associated with metabolic disturbances and aberrant inflammation signaling activation, and that identification of different ERS expression patterns could contribute to the precision treatment of OA patients. To identify ERS key regulators for enhancing the diagnosis and treatment of OA, we integrated two machine learning algorithms and PPI analysis to ensure the stability and robustness of our results. Ultimately, we identified two ERS key genes, IGFBP3 and S100A4, which exhibited excellent diagnostic performance for OA. In addition, we developed an ERS risk scoring system to predict the occurrence of OA, which demonstrated reliable clinical predictive and guidance benefits for OA patients.

IGFBP3 is a member of the insulin-like growth factor binding protein (IGFBP) family, which influence survival, apoptosis and differentiation by binding to specific receptors on the cell surface 38. The UPR transcription factor XBP1 has been shown to promote cell invasion by upregulating IGFBP3 expression 27. Pan et al. showed that IGFBP3 mediated ER stress-induced DNA damage by activating the PERK/eIF2α pathway 39. Additionally, Evans et al. found that IGFBP3 overexpression can induce cartilage catabolism and osteogenic differentiation and lead to the development of OA 40. In our study, both scRNA-seq and bulk RNA-seq identified IGFBP3 as a dangerous motor mediating ERS activation. We observed that knockdown of IGFBP3 could maintain cartilage metabolic homeostasis and inhibit cell apoptosis, thereby delaying OA development. ERS is intimately linked to cellular homeostasis and apoptosis 27. PERK is an important homeostatic monitoring protein on the ER. When sustained ERS occurs, GRP78 expression increases and dissociates from the PERK sensors thereby allowing their activation. The activation of PERK will lead to an increase in ATF4 transcription, inducing the expression of a variety of genes involved in the ERS response, including molecular chaperones and folding enzymes. Among these, CHOP is a direct downstream target gene of ATF4, which acts as a key apoptosis-inducing factor enabling ERS to shift from an adaptive to a pro-apoptotic response 27. S100A4, a member of the S100 protein family, is a calcium-binding protein that functions in motility, invasion, and microtubule protein polymerization 41. Overexpression of S100A4 can inhibit ERS and TLR4/NF-κB pathway to ameliorate apoptosis and inflammation 41. S100A4 has been found to be expressed highly in the synovium of OA and RA and can induce the expression and release of MMP-3 protein in synovial fluid 42. Altogether, these results improve novel thoughts and targets for regulating ERS in OA chondrocytes.

There are several limitations to this study to point out. First, it is mainly a retrospective study based on public databases with a relatively small sample size. As such, the findings need to be further validated by a multicenter prospective study. Furthermore, since the transcriptomic data used were derived from different microarray datasets, the stability of the results may be compromised, and thus further experiments and external cohort are required for validation.

## Conclusion

In this study, we utilized the collaborative advantages of scRNA-seq and Bulk RNA-seq to thoroughly investigate the complex role of ERS on chondrocyte in the development of OA. We revealed that ERS was involved in the progression and occurrence of OA by mediating metabolic and inflammation signaling pathways. Furthermore, ERS key regulators were developed and validated for the diagnosis and treatment of OA based on multiple bioinformatics approaches and experiments. Specifically, we found that the ERS regulator IGFBP3 activates the PERK/ATF4/CHOP cascade to trigger ERS to mediate chondrocyte apoptosis. These findings reveal the heterogeneity of ERS in OA and are of great significance for the understanding of the pathogenesis of OA and the development of personalized therapies. Further exploration of the specific mechanisms of ERS regulatory factors in OA and the development of effective targeted strategies will be the focus for future research.

## Supplementary Material

Supplementary figures and tables.
