# Supplementary material for: Decoding Endoplasmic Reticulum Stress on Chondrocyte Driving Osteoarthritis Development through Integrating Single-Cell and Transcriptomic Profiling
Source: Int J Med Sci. 2025 Aug 22;22(15):3906–23. doi: 10.7150/ijms.119573 (PMC12492377; doi:10.7150/ijms.119573)
Supplement: Supplementary file 1 — Supplementary figures and tables. [file ijmsv22p3906s1.pdf]

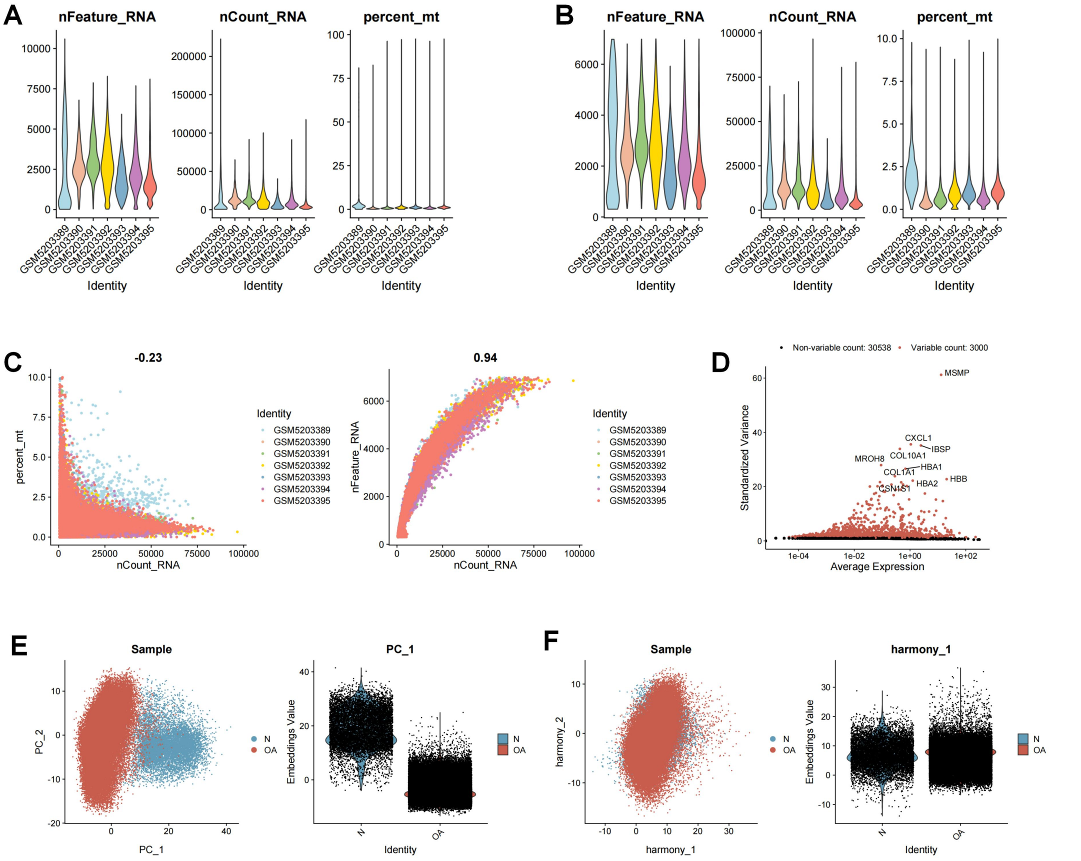

Figure S1. The quality control program of scRNA data. **(A-B).** Number of gene features, total counts of gene expression, and percentage of mitochondrial genes before and after processing; **(C).** The correlations of mitochondrial genes, gene counts, and gene features; **(D).** The top ten of highly variable genes; (E-F). Before and after removal of batch effects by the Harmony package.

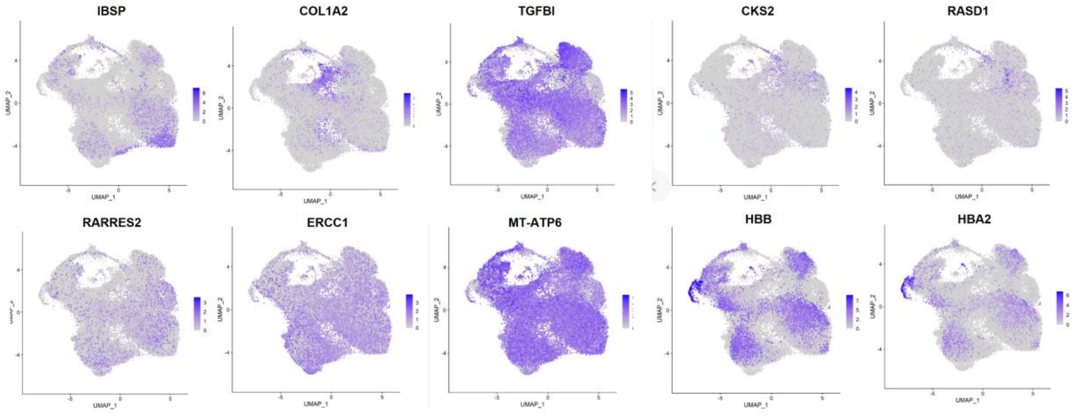

Figure S2. Marker genes for different subpopulation with UMAP.

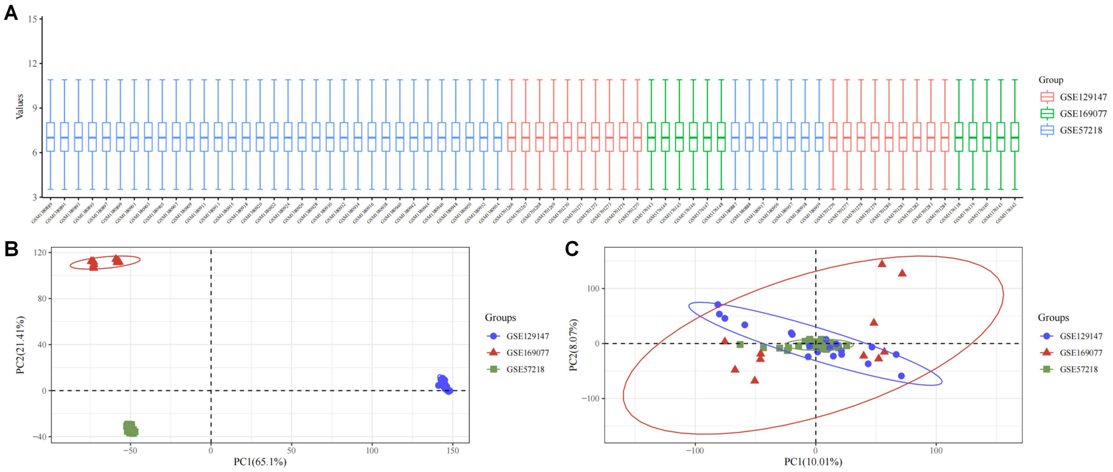

Figure S3. The pre-processing of bulk RNA-seq data. (A). Violin plots of the three GEO datasets integrated after removing batches; (B-C). PCA plots of before and after removing batches effect.

Supplementary Table 1. 295 unqiue ER stress-related genes obtained from MSigDB v7.4

PLA2G4B	FBXO27	BBC3	OPA1	PTPN1	ERMP1
BCL2L11	RNF183	SERP1	P4HB	PTPN2	DNAJB14
NR1H3	ATF6B	ERLEC1	PRKN	TRIB3	FOXRED2
CERT1	CREB3L4	AQP11	DERL2	BAK1	EDEM3
CTDSP2	CTH	RNF175	UBXN1	BAX	CDK5RAP3
PREB	DAB2IP	GRINA	TXNDC12	SRPRB	SEL1L2
PDIA6	CREBRF	PDIA3	RNFT1	SELENOK	UBXN6
BCAP31	TTC23L	GSK3A	BFAR	RASGRF1	CALR
LPCAT3	TMTC3	GSK3B	MBTPS2	RASGRF2	HM13
PIGBOS1	DCTN1	USP25	UBE2J1	CCND1	TMX1
TRIM13	DDIT3	POMT2	UFC1	BCL2	CANX
FLOT1	DDX3X	UBQLN2	JKAMP	BCL2L1	TMUB1
STUB1	DDX11	UBQLN1	UFM1	RNF5	SESN2
UBE4B	BHLHA15	NRBF2	GET4	CCL2	CASP4
MARCHF6	TOR1A	ERO1A	DNAJB11	SEL1L	ATG10
ABCA7	SCAMP5	HDGF	PIK3R1	TSPYL2	SRPX
RACK1	EEF2	CXXC1	PIK3R2	HERPUD2	TMEM117
CLGN	EIF2S1	UBE2K	PMAIP1	SGTA	RHBDD1
CREB3	EIF4G1	APAF1	PML	SHC1	NCK2
CEBPB	STT3B	DNAJB2	DNAJC10	PDIA2	SYVN1
HYOU1	DNAJC18	HSPA1A	TMCO1	CREB3L2	CREB3L3
AGR2	EP300	HSPA5	RNF186	DDRGK1	TMTC4
ERN2	ERN1	UBAC2	SGTB	BOK	PPP1R15B
ERLIN1	EXTL1	NCCRP1	DNAJB12	SRPRA	CAV1
CFTR	EXTL2	IGFBP1	PARP16	SSR1	MBTPS1
USP19	EXTL3	CXCL8	AUP1	SULT1A3	TNFRSF10B
YIF1A	FCGR2B	PDX1	FKBP14	TMBIM6	EIF2B5

SEC61B	SEC31A	ACADVL	WIPI1	THBS1	USP13
PDIA5	ATF6	ITPR1	ANKZF1	THBS4	BRSK2
SERINC3	ERP44	JUN	PPP2CB	TLN1	USP14
OS9	UBXN4	NHLRC1	TMEM33	TP53	CREB3L1
TMED2	FAF2	SERP2	PPP2R5B	HSP90B1	ATP6V0D1
ERP29	UFL1	LMNA	RNF121	TRAF2	TMEM67
COPS5	ECPAS	MIR200C	YOD1	UBE2G2	TMEM259
KDELR3	SIRT1	MAGEA3	NPLOC4	UFD1	AIFM1
FICD	TARDBP	MAN1A1	ARFGAP1	UMOD	DERL3
ERLIN2	PPP1R15A	DNAJB9	EDEM2	NR1H2	RNF185
RNF139	SDF2L1	MAP3K5	UGGT2	VCP	VAPB
MAN1B1	ALOX5	ATXN3	SELENOS	WFS1	TMEM129
PARK7	ALOX15	GET3	MYDGF	XBP1	UBE4A
FBXO17	ANKS4B	ASNS	DNAJC3	TRIM25	FBXO44
KLHDC3	SVIP	EIF2AK4	UGGT1	ZBTB17	EIF2AK3
NIBAN1	GORASP2	SULT1A4	CCDC47	RNF103	GOSR2
ADD1	FBXO2	ATF3	PSMC6	MANF	PDIA4
UBE2J2	FBXO6	ATF4	RCN3	TMUB2	EDEM1
CLU	FGF21	NCK1	RHBDD2	CHAC1	HERPUD1
TPP1	NUPR1	NFE2L2	PTPN1	DERL1	TATDN2
LRRK2	TBL2	ATP2A1	PTPN2	BAG6	CUL7
ERP27	AMFR	ATP2A2	TRIB3	UBA5	SEC16A
CALR3	GFPT1	ATP2A3	BAK1	UBXN8	

Supplementary Table 2. Canonical gene markers

**Cell subpopulations**	**Markers**
EC	TF,RARRES2
preHTC	STEAP1, TGFBI
FC	COL1A1,COL1A2
ProC	DDX21,ERCC1
RegC/HomC	JUN,TUBB4B,CKS2,RASD1
HTC	COL10A1,IBSP
MTC	MT-CYB,MT-ATP6
RBC	HBB,HBA1,HBA2

Table3. The primer sequences used in qPCR experiments.

Gene	Sequence
IGFBP3-F	CGTCTCCTGGAAACACCACT
IGFBP3-R	CAACCTGGCTTTCCACACTC
MMP3 -F	ACCTATTCCTGGTTGCTGCT
MMP3 -R	CAGGTCTGTGGAGGACTTGT
MMP9 -F	CCTGGAACTCACACAACGTC
MMP9 -R	TGCAGGAGGTCATAGGTCAC
MMP13 -F	CAGATTCTTCTGGCGTCTGC
MMP13 -R	CTCGGGATGGATGCTCGTAT
ADAMTS5 -F	ATGCAAGCATCGAGAACCAC
ADAMTS5 -R	GCTCACCTCCAGACTCTTGT
GAPDH-F	AGCCCTCCCTTCTCTCGAAT
GAPDH-R	CCCCACAACACTGCATTCAC
